# Citizens and conspiratorial anti-science beliefs: Opposition versus support in 38 countries across Europe

**DOI:** 10.1177/09636625241245371

**Published:** 2024-04-17

**Authors:** Joop de Boer, Harry Aiking

**Affiliations:** VU University, The Netherlands

**Keywords:** attitude toward vaccines, political (dis)satisfaction, science knowledge, social evaluations of scientists

## Abstract

This article aims to clarify citizens’ responses to conspiratorial anti-science beliefs (e.g. “The cure for cancer exists but is hidden from the public by commercial interests”). Based on Eurobarometer 95.2 (Spring 2021, 38 countries), we examine how public opposition or support for conspiratorial anti-science beliefs is related to individual- and country-level variables. There were large differences between the countries in their opposition or support. Controlling for artifacts, the individual-level variables showed associations with science-specific variables, for example, knowledge, preferred communication sources, social evaluations of scientists, attitude toward vaccines, and more general political (dis)satisfaction. At the country level, Affluence and Women’s representation were useful indicators for describing these differences. The conclusion is that the negativity of conspiratorial anti-science beliefs can be avoided by policies that highlight the rationality of science as a source of orientation and legitimation for change processes, and that are responsive to the needs of all citizens.

## 1. Introduction

In recent years, many countries have seen a destructive kind of criticism toward the world of science and certain individual scientists (e.g. Evans and Hargittai, 2020; Sanford et al., 2021). This phenomenon seems to combine two related categories of unwarranted beliefs and attitudes: That of conspiratorial ideas and that of anti-science attitudes (Enders et al., 2021; Priniski and Holyoak, 2022; Rizeq et al., 2021). The very existence and diffusion of conspiratorial anti-science (CAS) beliefs (e.g. “The cure for cancer exists but is hidden from the public by commercial interests”) highlights an opposition between “the common people” versus “the social elite,” including academics, scholars, and experts (Berman, 2019; Mede and Schäfer, 2020). The possible mistreatment of the people (e.g. “from whom the truth is hidden”) is combined with a misplaced image of science, which seriously contradicts the cultural values of intellectual autonomy, egalitarianism, and commitments to the welfare of others (Schwartz, 2014). This article examines the research question: How are European citizens’ opposition or support for CAS beliefs—after controlling for response style effects—related to science-specific knowledge, preferred communication sources, social evaluations of scientists and attitudes toward science products (e.g. vaccines), as well as general tendencies in European society (political (dis)satisfaction) and contextual differences between countries associated with indicators of national cultural values. The analysis is based on Eurobarometer 95.2 (conducted in Spring 2021, in 38 countries).

### Conceptualization of CAS beliefs

Citizens’ responses to CAS beliefs can be conceptualized in different ways. One option is that these beliefs are considered as a form of false information, the dissemination of which is closely connected with the rapid growth of social media users and platforms (Chen et al., 2023; Gwiaździński et al., 2023). In the words of Chen et al. (2023), this information category includes *misinformation* (false information, i.e. spread without the intent to mislead) and *disinformation* (false information, i.e. deliberately produced to cause harm), next to *fake news* (information that intends to deceive by mimicking the form of legitimate news). CAS beliefs might be spread to cause harm, but the current field of false information research is quite specific in its focus on online social interactions. Another option is that CAS beliefs are viewed as a form of epistemically unwarranted beliefs (Bensley et al., 2020), such as paranormal beliefs, belief in alternative medical practices; belief in pseudoscience and conspiracies. These beliefs are common among regular citizens and not pathological, but they are based on incorrect declarative knowledge, leading to suboptimal action (Oliver and Wood, 2014; Rizeq et al., 2021 Stanovich and Toplak, 2023). Although CAS beliefs are unwarranted, current research in this field is less relevant for science-specific topics, as it is often focused on general individual differences in reported thinking styles (Frenken and Imhoff, 2021; Pennycook et al., 2022; Swami et al., 2014).

A third option is considering opposition or support for CAS beliefs as responses to a rumor (i.e. an unconfirmed message about an object, person, or situation, passed from one person to another (Bordia and DiFonzo, 2005; Buckner, 1965; Moscovici, 2020)). This approach has many relevant aspects as it considers the information behavior of individuals in relation to their (online) social interactions and the substantive content of a message. Certain topics related to science and medicine belong to the traditional themes of rumors, such as rumors on the effects, origins, or cure for various ailments (Andrade, 2020; Enders et al., 2021). This illustrates that the message of a rumor may derive its importance from its connections with historical themes of a collective nature, associated with the value system and the culture of society, such as the theme of concealment (Moscovici, 2020) or manipulation by small powerful groups (Stoeckel and Ceka, 2023). Rumors related to science and medicine, which vary in importance over time and between countries, require continuous attention because they can create unwanted public health effects, such as in the case of the “vaccines cause autism” rumor community (Edy and Risley-Baird, 2016) or the COVID-19 vaccination rejection (Regazzi et al., 2023). The impacts of these rumors have given a new impetus to research on distinguishing factors that increase the likelihood of rejection of a rumor (if untrue) and factors that could lead to its further spread (see for a technological approach, Wu et al., 2023). Hence, rumor research has changed much over the past decades, partly as a result of developments in sociology (Pelletier et al., 2020), cognitive, motivational, and social psychology (Bordia and DiFonzo, 2005), and also as a result of changes in the world. For purposes of this analysis, it is useful to briefly consider the reception and sharing of rumors.

#### Reception and sharing of rumors

Regarding reception, Buckner (1965) already noted that individuals may adopt one of three orientations in relation to a rumor. The rumor, or the situation, may cause the individual to take *a critical stance, an uncritical stance*, or *a transmission stance* (just passing it on) toward it. If individuals take a critical stance, they can use their critical abilities to separate the true from the false in rumors they hear. If they also are knowledgeable about the subject matter of the rumor, they will be skeptical of its truth, unless the rumor fits in with what they knew before and they realize that sometimes rumors can be true (Buckner, 1965; Lee et al., 2021). In the words of cognitive oriented psychologists, both sufficient cognitive ability and the motivation to be critical are important in this context (Ståhl and Van Prooijen, 2018). Those who take an uncritical stance toward a rumor are unlikely to reject it, although they may transform it in their own way (Buckner, 1965). Certain circumstances and emotions hamper or eliminate the possibility of exercising critical ability. A key point is that citizens’ preferred information media can strongly affect their chances to be exposed to correct(ive) information (Scheufele and Krause, 2019); instead, they may receive too much incorrect information from their social network (Douglas et al., 2019; Pennycook et al., 2022).

The notion of rumor sharing puts the response to rumors in the broader context of social interchanges between persons and groups. Rumor sharing and further forms of social interchange may be guided by different motives, which in general terms can be described as the goal (1) to act effectively based on accurate knowledge, (2) to build and maintain relationships, and (3) to manage favorable self-impressions (Bordia and DiFonzo, 2005; Wood, 2000). The crucial point for rumor research is that only the first goal may reflect concerns with a valid understanding of reality; the other two goals may be served just as well by spreading a false rumor (Bordia and DiFonzo, 2005). One of the many consequences is that a person may share a particular rumor just to signal that he or she belongs to the group or category of people who have a very meaningful attitude toward the issue that is under discussion, such as members of a “rumor community” (Edy and Risley-Baird, 2016). Hence, in addition to the role of being knowledgeable for critical responses to the rumor, the person’s positive or negative attitude toward the subject matter becomes in particular important for rumor sharing in situations of ambiguity, danger or potential threat. For example, in a study that used two (hypothetical) rumors regarding food safety issues, Lee et al. (2021) found that their study participants’ negative attitude toward the food sources increased the likelihood of rumor sharing. In science-specific contexts, the attitude toward the subject matter relates to perceptions of scientists and science products.

#### Opposition or support for rumor-related beliefs

Factors connected to the reception and sharing of rumors are bound to influence, over time, the degree of opposition or support for specific rumor-related beliefs in a particular country (Enders et al., 2021; Oliver and Wood, 2014). Hence, comparing differences within and between countries in terms of their levels of opposition or support for CAS beliefs can provide important information about the development of these beliefs. Opposition or support may be related to science-specific variables, such as science knowledge or comprehension (Allum et al., 2008; Gauchat, 2011; Kahan, 2017), science communication sources (Scheufele and Krause, 2019), perceptions and social evaluations of scientists (Gligorić et al., 2022; Hendriks et al., 2015), and attitudes toward specific science products, such as vaccines (Andrade, 2020; Edy and Risley-Baird, 2016; Paul et al., 2021).

However, a special point of attention is that research into rumor-related beliefs is often based on questionnaire studies, which ask individuals whether they agree or disagree with a number of statements. This approach can inflate estimates of the prevalence of particular beliefs among the public, because a number of people tend to agree with such statements regardless of what they mean (acquiescence bias) (see, for an extended discussion of this topic, Oliver and Wood, 2014). The literature suggests that this response style is most prevalent in less (economically) developed and more (culturally) traditional countries (Rammstedt et al., 2017). As explained below, the biases can be reduced by adapting the question format (Clifford et al., 2019) and applying statistical controls for response tendencies (Bowes et al., 2021).

### The present study

#### Individual-level variables

This article considers the degree of opposition or support for CAS beliefs in 38 countries in relation to individual- and country-level variables. The individual-level variables were derived from Eurobarometer 95.2, aimed to measure “European citizens’ knowledge and attitudes toward science and technology” (European Commission, 2021b). The variables include the set of science-specific variables, mentioned above, which may reflect how individuals differently respond to statements that contain conspiratorial ideas and anti-science rhetoric. The analyses also included some more general variables, such as political (dis)satisfaction, length of education, religiosity, age, and gender. Growing dissatisfaction with democracy is considered to be a symptom or reflection of rising populism, emerging when citizens believe that political institutions are unwilling or unable to respond to their needs and demands (Berman, 2019). Length of education is important because it involves more than natural science knowledge and may also imply socialization experiences that relate to the transmission of the core values of a society (Meyer, 2015). The connections between religion-related variables and orientations toward science are complicated, but in Western countries, religiosity appears to be negatively associated with different orientations to science (Chan, 2018; Rutjens et al., 2022). There were no expectations on age and gender; the influence of gender depends on what aspect of science is under investigation (Evans and Hargittai, 2020).

#### Country-level variables

The survey data were complemented by country-level indicators that can show more comprehensive links with the prevailing values of the countries, such as “national income (per capita)” (henceforth, “Affluence”) and “the percentage of women seats in parliament and government” (henceforth, “Women’s representation”). These indicators reflect not only economic but also cultural differences between countries. This includes what the social psychologist Schwartz (2014, 2015) calls national cultural values, that is, conceptions of what is good and desirable among people in a society, which are manifested in the behavior of social institutions as well as individuals in relation to each other. In Schwartz’s theory, the core values reflect the importance of a person’s intellectual autonomy and moral equality, versus that of being embedded in existing social orders and traditions. Based on empirical data about 75 countries, Schwartz (2014, 2015) discusses the reciprocal causal processes that may account for the strong associations between, on one hand, their Affluence and other aspects of their socioeconomic level (including scientific activity), their level of democracy, and their level of Women’s representation and, on the other hand, the importance of autonomy and egalitarianism in the countries. Because the scores that Schwartz (2008) provides regarding intellectual autonomy, embeddedness and egalitarianism were only available for 31 countries in our study, it was decided to calculate (for illustrative purposes) their associations with Affluence and Women’s representation in these countries and to use the latter two variables subsequently as indicators for the values in all countries (see also de Boer and Aiking, 2023). Hence, a key point is that Affluence and Women’s representation refer to values that are supposed to be incongruent with CAS beliefs.

#### Main analysis

The main analysis was conducted using a multilevel modeling approach, stepwise adding individual- and country-level variables (Charlton, 2013). Key to the analysis is the comparison of two explanations of the country differences. One analysis shows (“bottom up”) how compositional differences associated with particular individual-level variables affect the degree of opposition or support for CAS beliefs in the countries. The other analysis describes (“top down”) how country-level variables specify the contextual differences in which opposing or supporting CAS beliefs in the countries can occur. Hence, it is particularly valuable to consider the role of the country-level variables with and without individual-level variables in the model.

#### Cross-level interactions

Due to the relative small number (38) of countries, cross-level interactions were only examined if there were theoretical reasons to expect differences (Bryan and Jenkins, 2016). Based on the literature, two potential cross-level interactions were identified. Both refer to the contextual factors that shape people’s practices. Noy and O’Brien (2019) demonstrate in a study among 41 countries that highly educated individuals in countries with high levels of scientific activity (and economic development) are more optimistic about science compared with their educational peers in countries where science is less central. Price and Peterson (2016) conclude in a study among 47 countries that the scientific gains that are brought by Affluence are accompanied by heightened fears of human-made risks. The first is relevant for correlations between CAS beliefs and length of education, the second for correlations between CAS beliefs and the attitude toward vaccines.

## 2. Method

### Individual-level data

Eurobarometer 95.2 was carried out in the 27 EU countries, plus five candidate countries (Albania, Montenegro, North Macedonia, Serbia, and Turkey), as well as Bosnia and Herzegovina, Kosovo, Iceland, Norway, Switzerland, and the United Kingdom. A description of the fieldwork for Eurobarometer research is included in the report of the European Commission (2021b). Due to the coronavirus pandemic, alternative interview modes to the usual computer-assisted personal interviews (CAPIs) were necessary in many countries. In these countries, participants were interviewed online, mostly after recruiting them in a probabilistic way by telephone. Potential recruitment or measurement mode effects were made visible in the analysis by a dummy variable. The study variables were taken from the archived data file (European Commission, 2021a). The text used in the questionnaire is presented in Supplemental List 1.

#### Dependent variable

The dependent variable is mean response to the two CAS items that were new in Eurobarometer 95.2 and that contained the statements about the cure for cancer and the production of viruses, respectively (for question wording, see Table 1). They were added to the set of nine true–false knowledge items on science topics (also in Table 1), which has been applied in many earlier studies (European Commission, 2005; National Science Board, 2016). This approach had several advantages for the measurement of CAS beliefs, response style, and scientific knowledge, which can be explained briefly. The participants were presented with a set of statements and asked to say whether they believed each statement to be true or false, with a “don’t know” response category to be used if they were unsure of their answer. The “don’t know” option is important to reduce response style effects (Clifford et al., 2019). In the present study, the nine knowledge items were also used to calculate to what extent the participants had a “true saying” response style (Bentler et al., 1971), which may be typical for knowledge items (Danner and Rammstedt, 2016). The measure of “true saying” was applied to control for response style effects on the responses to the CAS items.

**Table 1.** CAS beliefs and knowledge items: text, percent of correct and wrong answers in the pooled sample (*N* = 37,079), and inter-item correlations.

*CAS beliefs*	Correct answers (%)	Wrong answers (%)	Don’t know (%)	Item–rest correlation Set 1	Item–rest correlation Set 2
The cure for cancer exists but is hidden from the public by commercial interests. (False is correct.)	50	29	20		
Viruses have been produced in government laboratories to control our freedom. (False is correct.)	49	31	20		
*Knowledge items*					
The earliest humans lived at the same time as the dinosaurs. (False is correct.)	62	18	20	.38	.40
Antibiotics kill viruses as well as bacteria. (False is correct.)	54	34	12	.33	.38
Lasers work by focusing sound waves. (False is correct.)	41	24	35	.31	.33
The world’s human population is currently more than 10 billion. (False is correct.)	45	34	21	.29	.31
The continents on which we live have been moving for millions of years and will continue to move in the future. (True is correct.)	79	9	12	.35	.30
Human beings, as we know them today, developed from earlier species of animals. (True is correct.)	62	24	14	.29	.26
Climate change is for the most part caused by natural cycles rather than human activities. (False is correct.)	61	28	11	.21	.23
The oxygen we breathe comes from plants. (True is correct.)	82	11	6	.17	
The methods used by the natural sciences and the social sciences are equally scientific. (True is correct.)	46	27	27	.09	
Cronbach’s alpha				.57	.60
Mean inter-item correlation *r*				.13	.18

#### Independent variables

*Science knowledge* was based on the number of correct answers to the knowledge items.

The measures of *communication behavior* were based on several questions. The first question asked about the two main sources of information about developments in science and technology that the participants used (watched, read, or listened) the most. The list of eight items they could choose from included television (on a TV set or via the Internet), newspapers (either online or in print), online social networks and blogs (e.g. video hosting websites), and scientific journals or magazines (either online or in print). In addition, there were some questions on the frequency of Internet use (at home, on the place of work, on a mobile device), which were used to distinguish regular users from the other categories (non-regular users and non-users).

The *social evaluations of scientists* were derived from the responses to a list of ten characteristics that can be associated with scientists today. For each characteristic, the participants were asked to indicate if they think it describes scientists well or describes them badly. A special point of attention is that the list included both qualifying (“intelligent,” “reliable”) and disqualifying (“arrogant,” “narrow minded”) characteristics. This allows for measuring positive and negative evaluations independently, which is advisable if they are not completely opposite concepts (Lee et al., 2021).

The *attitude toward science products* was derived from the responses to an evaluative question on new technologies that are currently being developed. For each of them, the participants were asked whether they thought it will have a positive, a negative or no effect on our way of life in the next 20 years. The favorable or unfavorable evaluation of the technology “Vaccines and combatting infectious diseases,” (henceforth “attitude toward vaccines”) was chosen because this topic is more than the others related to the tradition of science and medicine rumors (Andrade, 2020; Paul et al., 2021) and, therefore, a potentially relevant correlate of CAS beliefs. As a control variable, the mean evaluation of the other technologies (solar energy/wind energy (combined), information and communication technology, brain and cognitive enhancement, biotechnology and genetic engineering, space exploration, nanotechnology, nuclear energy for energy production, and artificial intelligence) was used.

A measure of *political (dis)satisfaction* was derived from three questions. They referred to (dis)satisfaction with the way democracy works (in our country), (dis)agreement with the statement “my voice counts (in our country),” and the opinion that, in general, things are going in the right direction or in the wrong direction (in our country).

*Length of education* was based on the question how old the participants were when they stopped full-time education (categorized as at the age of 15 or below, 15–19 or 20 or above; those who were still studying were classified based on their current age).

The questions on *religiosity* referred (1) to the participants’ self-placement on a 10-point scale from “not at all religious or spiritual” to “very strongly religious or spiritual” and (2) to the faith they belonged to. The faith they mentioned was ordered into three categories: “no religion’’, “a religion that is not orthodox,” and “an orthodox religion.”

*Age* and *gender* were standard variable.

### Country-level data

The measure of *Affluence* was taken from the World Bank and refers to gross national income (GNI) per capita based on purchasing power parity (PPP) in constant 2017 international dollars, averaged over the years 2017–2020 (and log-transformed). *Women’s representation* was the percentage of seats held by women in national parliaments and governments (in Switzerland, only the parliament), taken from Eurostat and averaged over the past 5 years (2017–2021). For illustrative purposes, the cultural value orientation scores that Schwartz (2008) provides regarding intellectual autonomy, embeddedness, and egalitarianism, which were available for 31 of the 38 countries, were correlated with Affluence and Women’s representation (see Supplemental Table S1).

### Analyses

The preliminary analysis was used to condense the number of variables by considering patterns of related answers. This was applied to the questions on CAS beliefs, science knowledge, communication behavior, social evaluations of scientists, and political (dis)satisfaction. To assess the internal consistency of item sets, Cronbach’s alpha was calculated (Cronbach, 1951; Sijtsma, 2009). As the alpha-value tends to underestimate the internal consistency of scales consisting of fewer than 10 items, the average inter-item correlation was used as an alternative measure of internal consistency (Taber, 2018). This measure should fall in the range .15–.50, dependent on the generality (closer to .15) or specificity (closer to .50) of the target construct (Clark and Watson, 1995). Where the number of items is small, groupings of items were visually explored using multidimensional scaling (Proxscal). The results were also examined by means of bar charts.

The multilevel analysis with the averaged CAS items as the dependent variable was performed through linear mixed regression modeling, which considered individuals to be nested within countries. The analysis was performed in several models that stepwise added variables to the prediction (Charlton, 2013). As the variables were measured in quite different units, they were all standardized (*M* = 0 and *SD* = 1). The cross-level interactions were investigated by adding interaction terms to the regression model in which the relevant variables were multiplied (Jaccard and Turrisi, 2003). In these cases, the model included random slopes for the individual-level variables in the interaction term (Heisig and Schaeffer, 2019). As a check on the results, the countries were divided into three groups of about equal size: the groups represent the lowest levels, the medium levels, and the highest levels of Affluence. In addition, correlations were calculated between all the individual-level variables in the analysis. All calculations were made by SPSS 28 for Windows.

## 3. Results

### Checks on the variables

The first checks referred to the CAS beliefs, the number of “true saying” responses, and the knowledge scale. The responses to the two CAS items are presented in Table 1. Each CAS item was recoded into a 3-point scale (opposition = 0, don’t know = 0.5, support = 1). Their correlation in the pooled sample was *r* = .54 (*p* < .001), which indicates a strong association between specific constructs. The average number of “true” answers on nine knowledge items was four on a scale from zero to nine. The bar charts showed a curvilinear relationship between the number of “true” answers and support for the CAS beliefs, which increased among those who had a more than average number of “true” answers (see Supplemental Figure 1). To avoid quadratic terms, a new, more than average “true” saying index (starting with up to five times = 0) was created, which correlated *r* = .30 (*p* < .001) with the mean of the CAS items. Table 1 shows that about 30% of the pooled sample (*N* = 37,079) supported a CAS item; among those who did not give a more than an average number of “true” answers, this was about 24% (*N* = 30,162). Similarly, the opponents of the CAS items increased from 50% to 54% after the correction.

The knowledge items were recoded into 0 (wrong or don’t know) and 1 (correct). The reliability analysis of the nine items showed some weaknesses (Cronbach’s alpha = .57, mean intercorrelation = .13), which could be attributed to two items (see Table 1). One item (on plants as a source of oxygen) may have been too familiar, the other (on methods of the natural and the social sciences) was less factual than the others. After removal of the two weak items, the other items yielded a consistent scale (Cronbach’s alpha = .60, mean intercorrelation = .17), which agrees with their more general character as a broad measure of science knowledge.

The responses (maximal 2) to the eight items on sources of information about developments in science and technology were explored using multidimensional scaling (see Supplemental Figure 2). This analysis showed that television (63%), social media (29%), and newspapers (27%) had a separate position, whereas the five other options, including encyclopedia (14%), radio or podcasts (13%), and science journals (11%) were more interchangeable. Therefore, this information was condensed into four dummy variables: television, social media, newspapers, and other media. The questions on Internet use indicated that 87% participants were regular users. This information was also captured in a dummy variable.

The responses to the question on characteristics that can be associated with scientists today were coded into three categories (describes scientists badly, don’t know, and describes them well). The reliability analysis of the ten qualifying and disqualifying (reverse-coded) evaluations showed that two items had a negative correlation with the total scale: “altruistic” and “knows best what is good for people.” The two were not substantially correlated with each other (*r* = .21, *p* < .001) and could not form a reliable scale; they were thus not further analyzed. The remaining eight items yielded a reliable scale (Cronbach’s alpha = .72, mean intercorrelation = .24), but the mean intercorrelation was higher if the qualifying and disqualifying evaluations were kept separate. The four items “reliable,” “honest,” “collaborative,” and “intelligent” could be used for a measure of qualifying evaluations (Cronbach’s alpha = .66, mean intercorrelation = .33). The four items “arrogant,” “narrow minded,” “immoral,” and “bad at communication” could be used for a measure of disqualifying evaluations (Cronbach’s alpha = .64, mean intercorrelation = .31). The two evaluations correlated moderately negatively (*r* = -.37, *p* < .001) in the pooled sample. The control variable for the attitude toward vaccines was the mean evaluation of the other technologies (nine items, Cronbach’s alpha = .81, mean intercorrelation = .33).

The analysis of the three questions on political (dis)satisfaction yielded a Cronbach’s alpha of .69 (mean intercorrelation = .43). The responses to the item were transformed into *z*-scores and averaged. Table 2 gives an overview of all the variables.

**Table 2.** Description of the variables (before the *z*-score transformation).

Short variable name	Description	Minimum	Maximum	*M*	*SD*
*Individual level*					
CAS beliefs	Mean of the two CAS items	0	1	.40	.38
Interview mode	CAPI = 1	0	1	.57	.50
True saying	More than average “True saying” responses	0	4	.35	.87
Correct knowledge	Correct science knowledge (seven items)	0	7	4.24	1.74
Television	Communication behavior—television = 1	0	1	.63	.48
Social media	Communication behavior—social media = 1	0	1	.29	.46
Newspaper	Communication behavior—newspaper = 1	0	1	.26	.44
Other media	Communication behavior—other media = 1	0	1	.47	.50
Internet	Communication behavior—regular internet user = 1	0	1	.87	.33
Qualifying scientists	Qualifying evaluation of scientists (mean of four items)	1	3	2.54	.54
Disqualifying scientists	Disqualifying evaluation of scientists (mean of four items)	1	3	1.61	.59
Vaccines attitude	Positive or negative on vaccines	1	5	1.67	.78
Technology attitude	Positive or negative on other technologies (mean of nine items)	1	5	1.96	.49
Political dissatisfaction	Satisfied or dissatisfied with how democracy works (mean of three items)	1	5	2.29	.72
Length of education	Stopped at 15 or below, 15–19 or 20 or above	1	3	2.39	.64
Religiosity/spirituality	Self-placement on a 10-point scale	1	10	5.20	2.81
Orthodoxy	No religion, religion not orthodox, orthodox	1	3	2.06	.71
Age category	Age in six categories	1	6	3.80	1.64
Gender	Woman = 1	0	1	.52	.50
*Country level*					
Affluence	Gross national income (GNI) per capita in constant 2017 international dollars	11,033	80,059	38,921	16,742
Women’s representation	Percentage of seats held by women in national parliaments and governments	12	48	30	9

### Multilevel analysis

**Figure 1.**
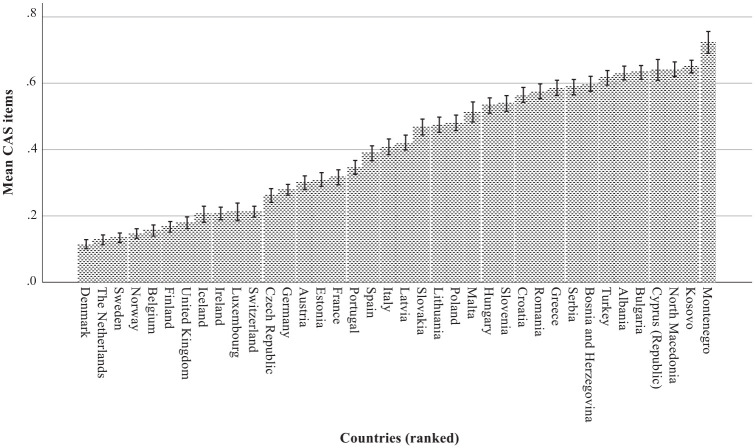
Mean proportion opposing (= 0) or supporting (= 1) CAS beliefs across countries, ranked from low to high (bar charts with 95% CI).

Figure 1 demonstrates that there were substantial differences between the countries in the degree to which the CAS beliefs were opposed or supported. The results of the multilevel analysis are reported in Table 3. The 0 model describes how the rejection of CAS beliefs differed between the countries. Due to the standardization of all variables, the mean value across countries was 0 and the variance 1. The between-country (level 2) variance is estimated as .238 and the within-country (individual level) variance is estimated as .769. The variance partition coefficient (VPC) is .236 (i.e. about 24% of the variance can be attributed to differences in intercept between the countries).

**Table 3.** CAS beliefs as the dependent variable in a multilevel regression analysis.^a^

	Model 0	Model 1	Model 2	Model 3	Model 4	Model 5	Model 6
*Regression coefficients and standard errors*							
Intercept	.016 (.079)	.003 (.072)	.067 (.059)	.018 (.047)	.018 (.032)	.028 (.031)	.012 (.033)
Interview mode		.023 (.024)	–.063 (.023)**	–.050 (.022)*	–.057 (.022)**	–.049 (.021)*	
True saying		.202 (.005)***	.194 (.004)***	.193 (.004)***	.192 (.004)***	.185 (.004)***	
Correct knowledge			–.156 (.005)***	–.140 (.005)***	–.139 (.005)***	–.137 (.005)***	
Television			.069 (.011)***	.062 (.011)***	.063 (.011)***	.057 (.011)***	
Social media			.087 (.011)***	.082 (.011)***	.082 (.011)***	.076 (.011)***	
Newspaper			–.096 (.012)***	–.071 (.012)***	–.070 (.012)***	–.065 (.012)***	
Other media			–.059 (.011)***	–.037 (.011)***	–.037 (.011)***	–.036 (.011)***	
Internet			–.037 (.014)**	–.012 (.015)	–.012 (.015)	–.006 (.015)	
Qualifying scientists			–.065 (.005)***	–.046 (.005)***	–.046 (.005)***	–.044 (.005)***	
Disqualifying scientists			.115 (.005)***	.108 (.005)***	.108 (.005)***	.107 (.005)***	
Vaccines attitude			.135 (.005)***	.119 (.005)***	.118 (.005)***	.126 (.012)***	
Technology attitude			.006 (.005)	–.007 (.005)	–.007 (.005)	–.002 (.005)	
Political dissatisfaction				.144 (.005)***	.143 (.005)***	.133 (.005)***	
Length of education				–.045 (.005)***	–.045 (.005)***	–.048 (.009)***	
Religiosity/spirituality				.034 (.005)***	.035 (.005)***	.035 (.005)***	
Orthodoxy				.054 (.007)***	.052 (.007)***	.046 (.007)***	
Age category				–.017 (.005)**	–.017 (.005)**	–.015 (.005)**	
Gender				.014 (.008)	.015 (.008)	.014 (.008)	
Affluence					–.163 (.024)***	–.180 (.022)***	–.357 (.034)***
Women’s representation					–.102 (.023)***	–.112 (.020)***	–.178 (.034)***
Affluence × higher education						–.022 (.008)*	
Affluence × vaccines attitude						.049 (.011)***	
*Covariance parameters*							
Residual	.769 (.006)	.733 (.005)	.645 (.005)	.626 (.005)	.626 (.005)	.617 (.005)	.769 (.006)
Intercept	.238 (.055)	.188 (.044)	.112 (.026)	.064 (.015)	.018 (.004)	.015 (.004)	.039 (.009)
UN (2,2)						.002 (.001)	
UN (3,3)						.004 (.001)	
*Information criteria model fit* ^b^							
–2 log-likelihood	95,686.120	93,874.557	89,145.266	87,976.529	87,928.431	87,505.998	95,618.548
Number of parameters	3	5	15	21	23	30	5

a All variables, except the dummy variables, have been standardized.

b The information criteria for model fit assessment are displayed in smaller-is-better form.

* *p* < .05, **p < .01, ***p < .001.

The Model 1 column includes the more than average “true”-saying tendency. This column shows that the between-country variance decreased from .236 to .188, which indicates that this tendency was partly related to country differences. The within-country variance decreased from .769 to .733. Model 2 presents the effects of the science-specific variables. A lower level of correct knowledge was associated with a higher support for CAS beliefs, but an inspection of the bar charts (not shown) revealed that those with zero correct answers did not have the highest support for CAS beliefs (see Supplemental Figure 3). Those for whom television and social media were important sources of information on science and technology developments showed somewhat more support for CAS beliefs; those who mentioned newspapers and other media showed more opposition. A qualifying evaluation of scientists was weakly associated with more opposition for CAS beliefs, but a disqualifying evaluation was somewhat stronger associated with more support. Controlling for the other variables, the attitude toward vaccines was also associated with more support for CAS beliefs. In this step, the between-country variance decreased from .188 to .112; the within-country variance decreased from.733 to .645.

The Model 3 column shows the associations with the more general individual-level variables. The first is political (dis)satisfaction, which had a relatively strong association with CAS beliefs. The other variables had small negative (longer education and higher age) or small positive (higher self-described religiosity or spirituality and a more orthodox religion) associations with support for CAS beliefs. Gender had no effect. The result of this analysis step is that the between-country variance decreased from .112 to .064, which was more than the decrease in the within-country variance from .645 to .626. The reduction in the between-country variance of Models 1, 2 and 3 suggests that this variance could be partly attributed to compositional effects (i.e. differences in the distribution of the individual-level variables between the countries).

**Figure 2.**
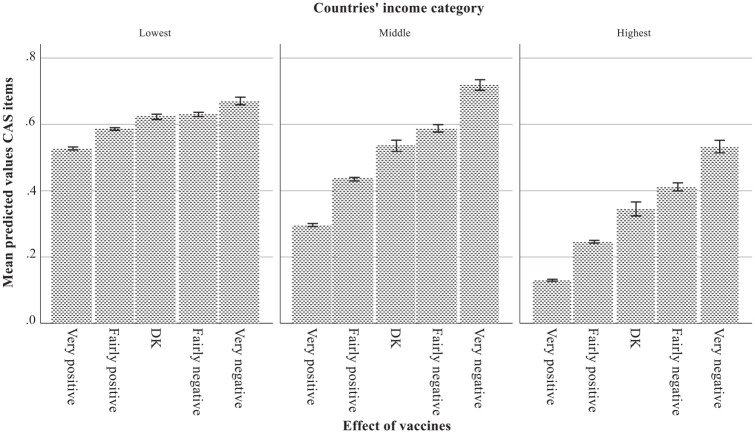
Predicted values of the CAS items with vaccines attitude by country’s income category (bar charts with 95% confidence intervals).

Model 4 demonstrates the additional contextual effects of Affluence and Women’s representation on the support for CAS beliefs in the countries; both were associated with more opposing responses. The between-country variance decreased from .064 to .018. Model 5 tested the cross-level interaction using Affluence as the level 2 variable. The negative relationship with length of education was not significant in lower-income countries, but small in higher-income countries (*B* = -.063, *SE* = .007), controlling for the other variables (including level of knowledge). The bar charts in Figure 2, using the predicted values of the regression model, show that the positive attitude toward vaccines had a stronger effect on opposing CAS beliefs in higher-income countries (*B* = .143, *SE* = .008) than in lower-income countries (*B* = .091, *SE* = .008).

Alternatively, to shed more light on the contextual and compositional effects of the country-level variables, Model 5 was run without the individual-level variables. The results of Model 6 indicate that Affluence and Women’s representation together reduced the between-country variance from .238 to .039, which is comparable to the reduction mentioned before due to compositional and contextual effects. Hence, in explaining country differences, the effects of the model parameters at the country level showed striking parallels with the compositional effects of the individual-level variables.

The correlations between the individual-level variables are presented in Supplemental Table S2. Finally, the complete set of independent variables was applied with as dependent variable the two CAS items, separately. The results show some small differences: vaccines attitude and political (dis)satisfaction had a slightly stronger associations with the item on viruses than with that on cancer; and the opposite applied to Women’s representation (see Supplemental Table S3).

## 4. Discussion

This study has provided valuable insights into the public’s responses to some conspiratorial, anti-scientific beliefs. The findings revealed that there were large differences between the 38 countries in their opposition or support for the two beliefs, ranging from Denmark, where 75% opposed both beliefs and 2% supported both beliefs, to Montenegro, where 16% opposed both beliefs and 59% supported both beliefs. The differences were partly a matter of “true” saying tendencies by participants. Controlling for this artifact, the differences were associated with a number of science-specific variables, such as level of science knowledge, preferred communication sources, social evaluations of scientists, and attitude toward vaccines. Also, more general variables, such as political (dis)satisfaction and, to a lesser extent, length of education, age, and religious variables played a role. In addition, there were contextual effects of country differences indicated by Affluence and Women’s representation. The expected cross-level interactions specified that length of education and the attitudes toward vaccines had larger associations with opposition or support for CAS beliefs in the higher-income countries.

In agreement with earlier work on citizens’ level of knowledge on science (Evans and Durant, 1995; Gauchat, 2011), our findings suggest that those displaying the lowest level may not be anti-science, but rather ambivalent toward it. Among them, the likelihood of opposing CAS beliefs was low but not completely absent. Among the others, the likelihood almost monotonously increased with higher numbers of correct answers, indicating that more knowledge tends to be associated with more concrete and often also more favorable attitudes toward science (Allum et al., 2008; Gauchat, 2011), except for controversial issues (Drummond and Fischhoff, 2017; Evans and Durant, 1995).

The preferred communication sources demonstrated the opposite associations of, on one hand, television, and social media, and on the other hand, newspapers, and other media. These differences may reveal different levels of motivations to be critical in the choice of media (see also Mede, 2022). It should be noted that regular Internet use and social media use were positively correlated, but that these variables had opposite relationships with responses to CAS beliefs: regular Internet use was negatively related with supporting CAS beliefs, social media use (as a source of information about developments in science and technology) positively. This agrees with recent literature on the positive association between use of social media as a source of information about COVID-19 and conspiracy beliefs (Allington et al., 2021).

The social evaluations of scientists and the attitude toward vaccines also had independent associations with responses to the CAS beliefs. Social evaluations of scientists may have become particularly important in the context of the COVID-19 pandemic, which dramatically increased the public visibility of certain scientists and highlighted their position as sources of practical information (Evans and Hargittai, 2020; Sanchez and Dunning, 2021). The qualifying evaluation of scientists may reflect, in different terms, what Hendriks et al. (2015) call, the “competence,” “adherence to scientific standards,” and “good intentions,” which are the grounds on which the public’s trust in science in general can be built (Wintterlin et al., 2022). Those who endorsed the qualifying evaluation of scientists were less likely to support CAS beliefs. In contrast, however, endorsing the disqualifying evaluation of scientists had a somewhat stronger and positive association with support for CAS beliefs, which may reflect the larger role of negativity in sharing negative rumors (Lee et al., 2021). Also, the analysis provided evidence for a specific association with the attitude toward vaccines (not with the other technologies); those who expected very positive or very negative effects had opposing or supporting responses to CAS beliefs, respectively.

The responses to the CAS beliefs were also related to the broader political and cultural context of the participants. The distinct role of political (dis)satisfaction was based on negative evaluations of topics that have no direct relationship with science. These evaluations may indicate how people felt that they were being treated, and a potential mistreatment of the people is an element of the CAS beliefs. In contrast, those participants who were very or fairly satisfied with how democracy works were more likely to oppose the CAS beliefs.

The contextual effects of country differences in Affluence and Women’s representation may demonstrate that the CAS beliefs present an image of science that seriously contradicts the cultural values of intellectual autonomy (key specific values: curiosity, broadmindedness, creativity) and egalitarianism (key values: equality, social justice, responsibility, honesty) (Schwartz, 2014, 2015). The effects of more years of education were somewhat larger in higher-income countries, which agree with the suggestion by Noy and O’Brien (2019) that an increased positive relationship between education and science attitudes reflects a country’s greater emphasis on the values of, what they call, “rationality and individualism.” The specific association with the attitude toward vaccines was also larger in higher-income countries. Price and Peterson (2016) mentioned heightened fears of human-made risks in affluent countries. In the higher-income countries, there were more participants who expected positive effects of vaccines and Figure 2 reveals that they were more often than others opposed to CAS beliefs, which may reflect their recent experiences and is in line with their cultural values. Yet, there was support for CAS beliefs from those who anticipated negative effects.

### Limitations

The main limitation of our approach is its dependence on a secondary analysis of survey data, which means that the work is limited to the questions asked by the original investigators, guided by EU policy development. On one hand, this is a strength because it might be assumed that the questions are policy relevant (Haverland et al., 2018). However, it is a weakness in that the set of variables cannot shed more light on all the variables with a specific relevance in this context. Another weakness is that we did not have direct evidence on the role of national cultural values. The cultural value scores that Schwartz (2008) calculated were not available for all the countries in our study.

## 5. Conclusion

The negativity involved in CAS beliefs and the threats to science that have been reported in the literature make it important to examine the relationships between citizens’ responses to CAS beliefs and a range of science-specific variables and more general tendencies in European society. The results showed large differences between the countries in opposing and supporting CAS beliefs. The analysis revealed that Affluence and Women’s representation were useful indicators for describing these differences, which suggest the influence of particular cultural value orientations. These value orientations at the country level may have found expression in the individual-level variables that accounted for compositional effects in explaining the country differences. Controlling for artifacts, the individual-level variables demonstrated distinct associations between, on one hand, opposition or support of CAS beliefs, and on the other hand, level of science knowledge, preferred communication sources, social evaluations of scientists, and attitude toward a specific science product (e.g. vaccines), together with political (dis)satisfaction, length of education, and religiosity. Additional contextual differences at the country level were also related to Affluence and Women’s representation.

What the results have in common is that they reveal that CAS beliefs were either incongruent or congruent with certain cultural value orientations that differed between individuals and countries. Cultural value orientations make rumors or beliefs that are compatible with them seem natural and give legitimacy to attempts to reject rumors or beliefs that contradict prevailing values. Although the analysis does not provide direct evidence on what these orientations are, there are theoretical and empirical reasons to assume that they may refer to the cultural importance of intellectual autonomy and egalitarianism, which are incongruent with CAS beliefs. A case in point is that the association between opposing or supporting CAS beliefs and vaccines attitude showed a different profile in higher- than in lower-income countries. In the higher-income countries, there were more participants who expected positive effects of vaccines and they demonstrated a stronger opposition to CAS beliefs. Nevertheless, there were also participants who anticipated negative effects and supported CAS beliefs, often in association with political dissatisfaction. The results underline the importance that many citizens have an adequate level of scientific literacy to understand what’s going on in the world. However, their attitudes and cultural values are also important. Countries where the opposition to CAS beliefs is low are vulnerable to the consequences of future exposure to false information. For a number of countries across Europe, there is reason for concern about this prospect. Although the evidence is correlational, the negativity mentioned in the literature can be avoided by consistent policies and actions that highlight the rationality of science as a source of orientation and legitimation for change processes, and that are responsive to the needs of all citizens.

## Supplemental Material

sj-docx-1-pus-10.1177_09636625241245371 – Supplemental material for Citizens and conspiratorial anti-science beliefs: Opposition versus support in 38 countries across Europe Supplemental material, sj-docx-1-pus-10.1177_09636625241245371 for Citizens and conspiratorial anti-science beliefs: Opposition versus support in 38 countries across Europe by Joop de Boer and Harry Aiking in Public Understanding of Science
