# Supplementary material for: Citizens and conspiratorial anti-science beliefs: Opposition versus support in 38 countries across Europe
Source: Public Underst Sci. 2024 Apr 17;33(8):1027–45. doi: 10.1177/09636625241245371 (PMC11505401; doi:10.1177/09636625241245371)
Supplement: sj-docx-1-pus-10.1177_09636625241245371 – Supplemental material for Citizens and conspiratorial anti-science beliefs: Opposition versus support in 38 countries across Europe [file sj-docx-1-pus-10.1177_09636625241245371.docx]

#### Citizens and Conspiratorial Anti-Science Beliefs: Opposition vs Support in 38 Countries across Europe

Joop de Boer *

Harry Aiking

Institute for Environmental Studies, VU University, Amsterdam, The Netherlands

Supplemental Material

Table of Contents

Supplemental List 1. Text of the items used in the questionnaire 2

Supplemental Table S1. Correlations between the country-level variables Affluence and Women’s representation and Schwartz’s cultural values indicators6

Supplemental Figure 1. Mean of the two CAS items with number of true saying answers 7

Supplemental Figure 2. Positions of the sources of information about developments in science and technology in the multidimensional space  8

Supplemental Figure 3. Mean of the two CAS items with number of correct knowledge answers 9

Supplemental Table S2. Correlations between the individual-level variables 10

Supplemental Table S3. Using the CAS beliefs separately as dependent variables in the multilevel regression analysis 11

* Address for correspondence:

Joop de Boer, Institute for Environmental Studies, VU University, De Boelelaan 1111, 1081 HV Amsterdam, The Netherlands, E-mail: joop.de.boer@vu.nl

Supplemental List 1. Text of the items used in the questionnaire

Source: Eurobarometer 95.2 (April-May 2021) Basic bilingual questionnaire ZA7782. Kantar Public, Brussels; GESIS, Cologne.

For each of the following statements, please indicate whether you believe them to be true or false. If you don't know, you can just indicate so.

(ROTATE RESPONSE LIST)

The earliest humans lived at the same time as the dinosaurs

The continents on which we live have been moving for millions of years and will continue to move in the future

Antibiotics kill viruses as well as bacteria

The oxygen we breathe comes from plants

Lasers work by focusing sound waves

The world's human population is currently more than 10 billion

The methods used by the natural sciences and the social sciences are equally scientific

Human beings, as we know them today, developed from earlier species of animals

Climate change is for the most part caused by natural cycles rather than human activities

The cure for cancer exists but is hidden from the public by commercial interests

Viruses have been produced in government laboratories to control our freedom

Of the following list of sources of information about developments in science and technology, please choose the two main sources that you use (watch, read, or listen) the most.

(ROTATE RESPONSE LIST / MAX. 2 ANSWERS)

Television, on a TV set or via the internet

Newspapers, either online or in print

Online encyclopaedias e.g. Wikipedia

Magazines, either online or in print

Radio, including podcasts

Books, either in print or e-books

Online social networks and blogs (e.g. video hosting websites)

Scientific journals, either online or in print

The following is a list of characteristics that can be associated with scientists today. For each characteristic, indicate if you think it describes scientists well or describes them badly

(RANDOMISE RESPONSE LIST)

Reliable

Collaborative

Narrow minded

Bad at communicating

Honest

Arrogant

Altruistic

Immoral

Intelligent

Know best what is good for people

The following is a list of areas where new technologies are currently being developed. For each of these, do you think it will have a positive, a negative or no effect on our way of life in the next 20 years?

(ROTATE RESPONSE LIST)

Solar energy

Wind energy

Information and communication Technology

Brain and cognitive enhancement

Vaccines and combatting infectious diseases

Biotechnology and genetic engineering

Space exploration

Nanotechnology

Nuclear energy for energy production

Artificial Intelligence

On the whole, are you very satisfied, fairly satisfied, not very satisfied or not at all satisfied with the way democracy works in (OUR COUNTRY)?

Very satisfied

Fairly satisfied

Not very satisfied

Not at all satisfied

Don't know

To what extent do you agree or disagree with each of the following statement

My voice counts in (OUR COUNTRY)

Totally agree

Tend to agree

Tend to disagree

Totally disagree

Don't know

At the present time, would you say that, in general, things are going in the right direction or in the wrong direction, in…(OUR COUNTRY)?

Things are going in the right direction

Things are going in the wrong direction

Don't know

How old were you when you stopped full-time education?

The answers were categorized as (1) at the age of 15 or below, (2) 15-19 or (3) 20 or above; those who were still studying were classified based on their current age.

On a scale of 1 to 10, how religious or spiritual do you consider yourself? 1 means that you are not at all religious or spiritual, 10 that you are very strongly religious or spiritual. The remaining numbers indicate something between these two positions.

1 Not at all religious or spiritual

2

3

4

5

6

7

8

9

10 Very strongly religious or spiritual

Refusal

Don't know

Do you consider yourself to be…

1 Catholic

2 Orthodox Christian

3 Protestant

4 Other Christian

5 Jewish

6 Muslim – Shia

7 Muslim – Sunni

8 Other Muslim

9 Sikh

10 Buddhist

11 Hindu

12 Atheist

13 Non-believer or agnostic

14 Other

Refusal

Don't know

Codes: 1, 3, 5, 10, 11, 14 were considered “a religion that is not orthodox” and 2, 6, 7, 8, 9 were considered “an orthodox religion”.

Supplemental Table S1. Correlations between the country-level variables Affluence and Women’s representation and Schwartz’s cultural value orientation scores)

|  | 1 | 2 | 3 | 4 |
| --- | --- | --- | --- | --- |
| 1 Affluence |  |  |  |  |
| 2 Women’s representation | .48 |  |  |  |
| 3 Intellectual autonomy | .54 | .58 |  |  |
| 4 Embeddedness | -.73 | -.63 | -.83 |  |
| 5 Egalitarianism | .65 | .54 | .54 | -.71 |

(*N* = 31)

*Intellectual autonomy* encourages individuals to pursue their own ideas and intellectual directions independently.

In *embeddedness* cultures, people are viewed as entities embedded in the collectivity. Meaning in life is expected to come largely through social relationships, through identifying with the group, participating in its shared way of life, and striving toward its shared goals. Embedded cultures emphasize maintaining the status quo and restraining actions that might disrupt in-group solidarity or the traditional order.

*Egalitarian* cultures seek to induce people to recognize one another as moral equals who share basic interests as human beings. They try to socialize their members to internalize a commitment to cooperate and to feel concern for everyone's welfare. People are expected to act for the benefit of others as a matter of choice.

^1^) Source: Schwartz SH (2008) The 7 Schwartz cultural value orientation scores for 80 countries. Data file. <https://www.researchgate.net/publication/304715744_The_7_Schwartz_cultural_value_orientation_scores_for_80_countries>. Accessed 1 July 2022.

Supplemental Figure 1. Mean of the two CAS items with number of true saying answers (Error bars with 95% confidence interval).

Supplemental Figure 2. Positions of the sources of information about developments in science and technology in the multidimensional space (Model = interval, Normalized Raw Stress = .006, *N* = 37,079)

Supplemental Figure 3. Mean of the two CAS items with number of correct knowledge answers (Error bars with 95% confidence interval).

Supplemental Table S2. Correlations between the individual-level variables

|  | 1 | 2 | 3 | 4 | 5 | 6 | 7 | 8 | 9 | 10 | 11 | 12 | 13 | 14 | 15 | 16 | 17 |
| --- | --- | --- | --- | --- | --- | --- | --- | --- | --- | --- | --- | --- | --- | --- | --- | --- | --- |
| 1 CAS beliefs |  |  |  |  |  |  |  |  |  |  |  |  |  |  |  |  |  |
| 2 True saying | .30 |  |  |  |  |  |  |  |  |  |  |  |  |  |  |  |  |
| 3 Correct knowledge | -.37 | -.18 |  |  |  |  |  |  |  |  |  |  |  |  |  |  |  |
| 4 Television | .08 | .02 | -.12 |  |  |  |  |  |  |  |  |  |  |  |  |  |  |
| 5 Social media | .12 | .06 | -.01 | -.18 |  |  |  |  |  |  |  |  |  |  |  |  |  |
| 6 Newspaper | -.21 | -.10 | .15 | -.08 | -.25 |  |  |  |  |  |  |  |  |  |  |  |  |
| 7 Other media | -.15 | -.09 | .19 | -.41 | -.23 | -.22 |  |  |  |  |  |  |  |  |  |  |  |
| 8 Internet | -.14 | -.03 | .22 | -.12 | .19 | .08 | .12 |  |  |  |  |  |  |  |  |  |  |
| 9 Qualifying scientists | -.17 | .05 | .14 | .02 | .02 | .05 | .03 | .08 |  |  |  |  |  |  |  |  |  |
| 10 Disqualifying scientists | .22 | .05 | -.14 | -.01 | .00 | -.06 | -.03 | -.09 | -.37 |  |  |  |  |  |  |  |  |
| 11 Vaccines attitude | .28 | .04 | -.19 | -.04 | .05 | -.12 | -.04 | -.09 | -.30 | .21 |  |  |  |  |  |  |  |
| 12 Technology attitude | .15 | -.05 | -.19 | .01 | -.07 | -.05 | -.07 | -.19 | -.34 | .21 | .53 |  |  |  |  |  |  |
| 13 Political dissatisfaction | .30 | .06 | -.13 | .02 | .09 | -.15 | -.08 | -.08 | -.18 | .11 | .22 | .14 |  |  |  |  |  |
| 14 Length of education | -.23 | -.09 | .30 | -.14 | .06 | .14 | .17 | .36 | .13 | -.13 | -.13 | -.18 | -.13 |  |  |  |  |
| 15 Religiosity/ spirituality | .25 | .08 | -.31 | .09 | .01 | -.11 | -.11 | -.18 | -.07 | .08 | .12 | .13 | .06 | -.19 |  |  |  |
| 16 Orthodoxy | .35 | .16 | -.38 | .09 | .08 | -.18 | -.16 | -.16 | -.09 | .08 | .15 | .10 | .13 | -.21 | .58 |  |  |
| 17 Age category | -.06 | -.05 | -.04 | .21 | -.33 | .14 | -.04 | -.32 | -.06 | .04 | -.06 | .09 | -.03 | -.17 | .08 | -.04 |  |
| 18 Gender | .04 | .00 | -.14 | .04 | .01 | -.02 | -.04 | -.01 | .00 | -.02 | .02 | .07 | .00 | -.03 | .13 | .04 | -.03 |

Supplemental Table S3. Using the CAS beliefs separately as dependent variables in the multilevel regression analysis

|  | CAS belief on cancer | |  | CAS belief on viruses | |
| --- | --- | --- | --- | --- | --- |
| *Regression coefficients and standard errors* | B | SE |  | B | SE |
|  |  |  |  |  |  |
| Intercept | .041 | .032 |  | -.007 | .032 |
| Interview mode | -.072 | .023 |  | -.012 | .022 |
| True saying | .148 | .005 |  | .177 | .005 |
| Correct knowledge | -.117 | .005 |  | -.123 | .005 |
| Television | .037 | .012 |  | .063 | .011 |
| Social media | .063 | .012 |  | .072 | .012 |
| Newspaper | -.065 | .013 |  | -.050 | .012 |
| Other media | -.028 | .012 |  | -.035 | .012 |
| Internet | -.010 | .016 |  | -.001 | .015 |
| Qualifying scientists | -.043 | .005 |  | -.034 | .005 |
| Disqualifying scientists | .095 | .005 |  | .093 | .005 |
| Vaccines attitude | .101 | .010 |  | .120 | .012 |
| Technology attitude | -.003 | .006 |  | .000 | .006 |
|  |  |  |  |  |  |
| Political dissatisfaction | .102 | .005 |  | .132 | .005 |
| Length of education | -.041 | .009 |  | -.043 | .008 |
| Religiosity/ spirituality | .030 | .006 |  | .033 | .006 |
| Orthodoxy | .032 | .007 |  | .050 | .007 |
| Age category | -.018 | .005 |  | -.009 | .005 |
| Gender | .027 | .009 |  | -.003 | .009 |
|  |  |  |  |  |  |
| Affluence | -.151 | .021 |  | -.164 | .022 |
| Women’s representation | -.127 | .020 |  | -.069 | .020 |
|  |  |  |  |  |  |
| Affluence*Higher education | -.022 | .009 |  | -.018 | .008 |
| Affluence*Vaccines attitude | .047 | .010 |  | .040 | .011 |
|  |  |  |  |  |  |
|  |  |  |  |  |  |
|  |  |  |  |  |  |
| *Covariance parameters* |  |  |  |  |  |
| Residual | .722 | .005 |  | .676 | .005 |
| Intercept | .013 | .003 |  | .015 | .004 |
| UN (2,2) | .002 | .001 |  | .002 | .001 |
| UN (3,3) | .003 | .001 |  | .004 | .001 |
